# Histopathology imagery dataset of Ph-negative myeloproliferative neoplasm

**DOI:** 10.1016/j.dib.2023.109484

**Published:** 2023-08-11

**Authors:** Umi Kalsom Mohamad Yusof, Syamsiah Mashohor, Marsyita Hanafi, Sabariah Md Noor, Norsafina Zainal

**Affiliations:** a Department of Computer and Communication Systems Engineering, Faculty of Engineering, Universiti Putra Malaysia, 43400 UPM, Serdang, Selangor, Malaysia; b Department of Pathology, Faculty of Medicine and Health Sciences, Universiti Putra Malaysia, 43400 UPM, Serdang, Selangor, Malaysia; c Pathology Department, Hospital Serdang, Jalan Puchong, 43000 Kajang, Selangor, Malaysia

**Keywords:** Myeloproliferative neoplasm, Polycythaemia vera, Essential thrombocythemia, Primary myelofibrosis, Machine learning, Artificial intelligence

## Abstract

Tumorous cancer has been a widely known and well-studied medical phenomenon; however, rare diseases like Myeloproliferative Neoplasm (MPN) have received less attention, leading to delayed diagnosis. Despite the availability of advanced technology in diagnostic tools that can boost the procedure, the morphological assessment of bone marrow trephine (BMT) images remains critical to confirm and differentiate MPN subtypes. This paper reports a histopathological imagery dataset that was created to focus on the most common MPN from the Philadelphia Chromosome (Ph)-negative type, namely Essential Thrombocythemia (ET), Polycythemia Vera (PV), and Primary Myelofibrosis (MF). The dataset consisted of 300 BMT images that can be used to enable computer vision applications, such as image segmentation, disease classification, and object recognition, in assisting the classification of the MPN disease. Ethical approval was obtained from the Ministry of Health, Malaysia and the bone marrow trephine images were captured using a digital microscope from the Olympus model (BX41 Dual head microscope) with x10, x20, and x40 lens types. The development of comprehensive tools deployed from this dataset can assist medical practitioners in diagnosing diseases, thus overcoming the current challenges.

**Specifications Table**

**Table:** 

Subject	Applied Machine Learning, Biomedical Engineering
Specific subject area	Computer-aided vision for the classification of Ph-Negative Myeloproliferative Neoplasm specifically for Essential Thrombocythemia (ET), Polycythemia Vera (PV), and Primary Myelofibrosis (MF).
Data format	Raw data consisting of histopathology images having RGB colour in PNG format
Type of data	Raw data
Data collection	Bone marrow biopsy images of MPN patients with confirmed diagnosis by the pathologist and physician were collected from Hospital Serdang, Malaysia. Ethical approval was obtained from the Ministry of Health, Malaysia with reference number NMRR-18-4023-42507 (IIR) upon data collection. The BMT images were captured using a digital microscope from the Olympus model (BX41 Dual head microscope) with lens types x10, x20, and x40. In total, 300 bone marrow biopsy images were captured from 11 raw slides provided by the haematologist. This data collection process had no direct interaction between the data collector and the patient. Medical data used in this research were anonymous and no personal details of the patients were required or gathered in the data collection procedure.
Data source location	Institution: Hospital Serdang, Malaysia City/Town/Region: Serdang Country: Malaysia
Data accessibility	Repository name: Mendeley Data Data identification number: doi: 10.17632/hbdh66ws8d.1 Direct URL to data: https://data.mendeley.com/datasets/hbdh66ws8d/1

## 1 Value of the Data

• Bone marrow morphology is of fundamental importance to distinguish different Myeloproliferative Neoplasm (MPN) subtypes [1], [2], [3]. This MPN dataset is able to assist medical practitioners, particularly in familiarising them with the morphology features with good qualitative image analysis.
• With proper augmentation methods, the MPN dataset can be expanded and used to train machine learning models for the classification of Philadelphia Chromosome (Ph)-Negative MPN.
• The MPN dataset can be utilised as a test dataset or validation in the machine learning classification model.
• Additionally, the MPN dataset can be used by machine learning researchers or data scientists to develop solutions using artificial intelligence techniques for addressing problems such as human error, high dependency on human expertise, interobserver variability, and delayed diagnosis [2], [3], [4].
• The generated dataset can be used to facilitate various computer vision tasks such as image segmentation, disease classification, and object recognition.

## 2 Data Description

This imagery dataset consisted of 300 labelled histopathological images from three common types of Ph-Negative MPN, namely Essential Thrombocythemia (ET), Polycythemia Vera (PV), and Primary Myelofibrosis (MF). All images were in the RGB format and the type of extension images was standardised to ‘.png’. The Portable Network Graphics, or PNG format, was selected because it has the property of lossless images when processing occurs, such as decreasing the file size.

**Fig. 1:**
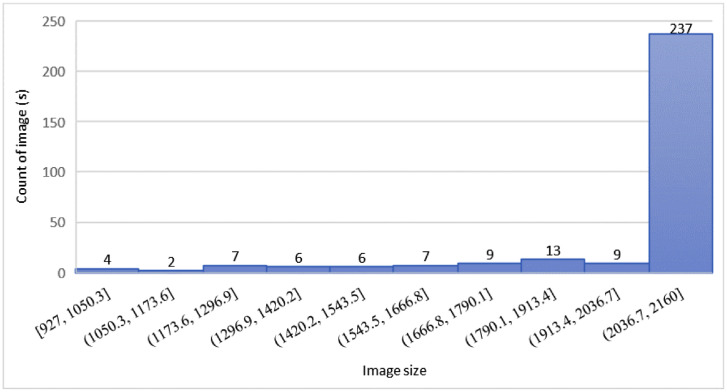
Distribution of image size was skewed to the right in the MPN dataset.

The distribution of image size in the dataset was prepared as in Fig. 1. This information can guide users to justify the appropriate input size in image preparation during the machine learning model development since image resolutions may vary. The data distribution was observed skewed to the right where a larger image size was found in the highest number. In total, the largest size of images in the dataset was distributed between 2036 × 2036 pixels and 2160 × 2160 pixels with 237 images, while the smallest size was found between 927 × 927 pixels and 1050.3 × 1050.3 pixels with 4 images.

**Fig. 2:**
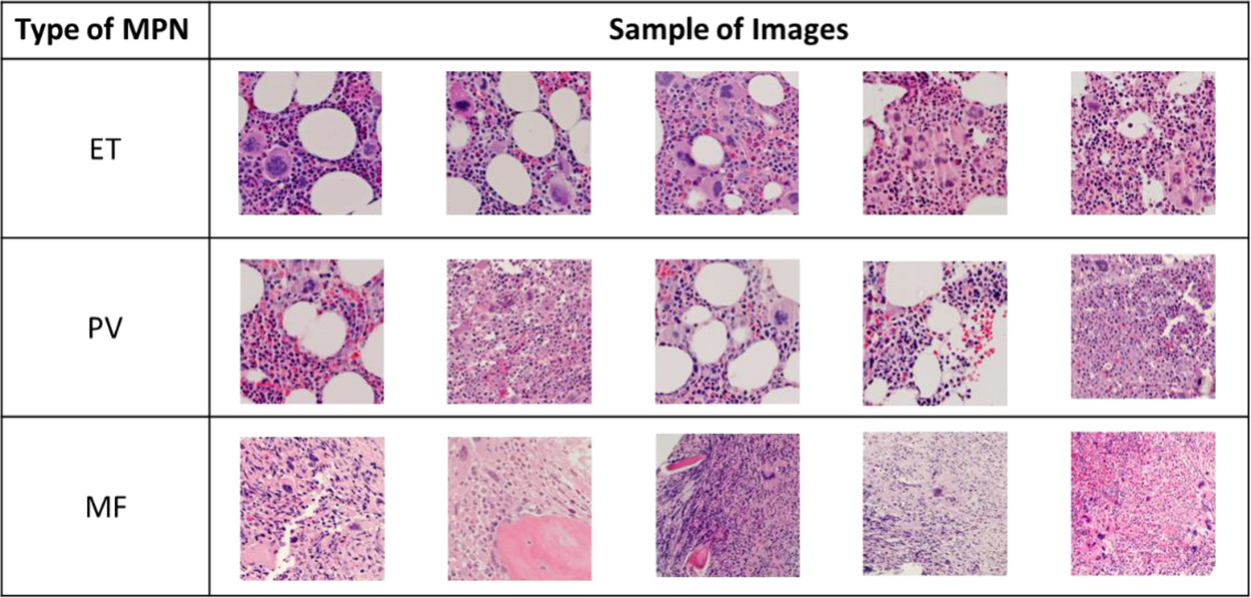
Sample images for ET, PV, and MF in the imagery dataset.

Following the ethical guideline, the images collected were confirmed as anonymous and did not carry any personal information related to the patients in the documentation. The samples of images in the dataset are shown in Fig. 2.

Adopted from Thiele et al., Table 1 was provided to conclude the BMT morphological criteria in the dataset and relative frequency of features for PV, ET, and MF [5].

**Table 1:** Relative frequency of features in bone marrow for PV, ET, and MF [5].

## 3 Experimental Design, Materials, and Methods

### 3.1 Data acquisition

**Fig. 3:**
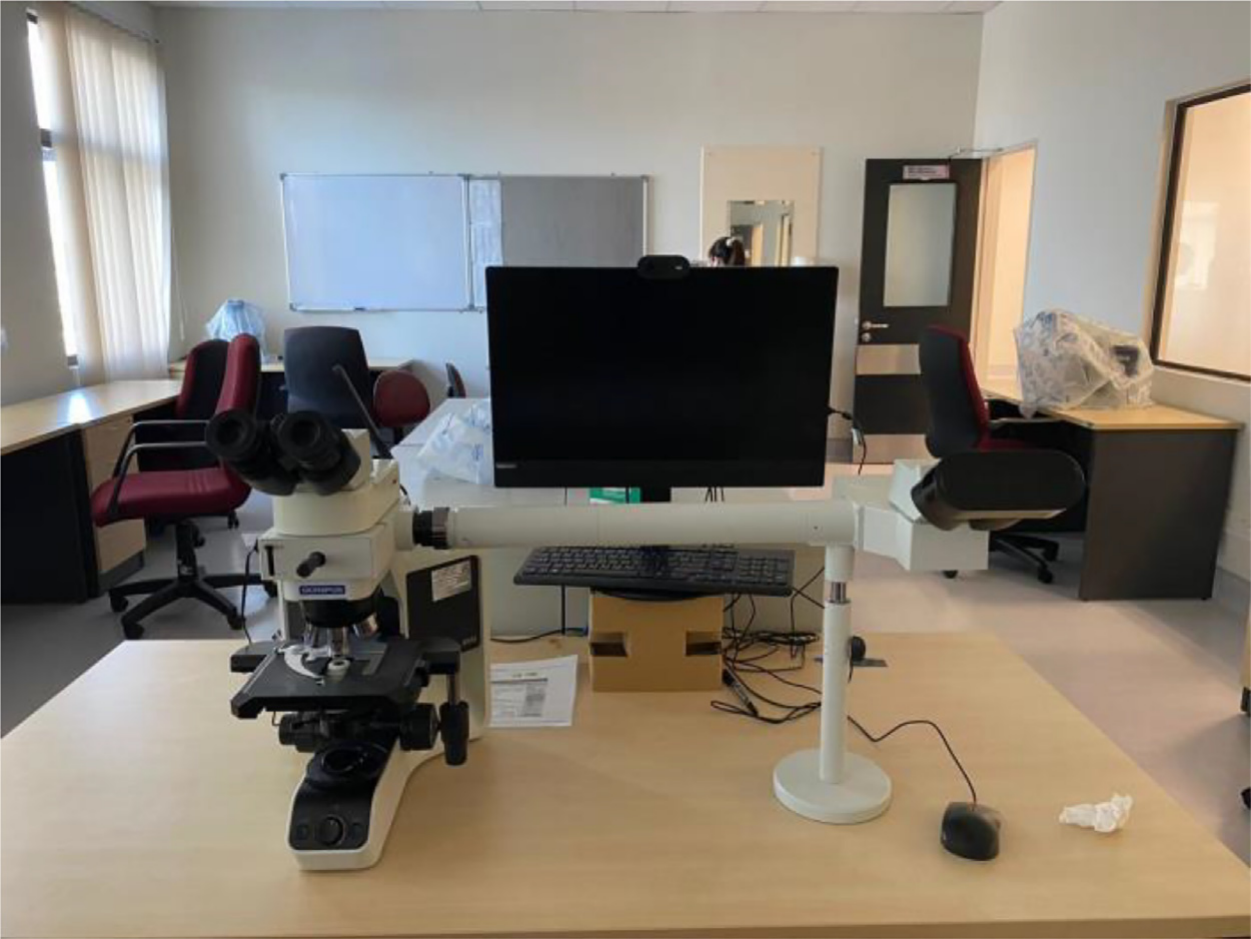
Olympus BX41 Dual Viewing Side-By-Side Microscope.

The sample of bone marrow biopsy of patients with a confirmed diagnosis of MPN was prepared by the pathologist at Hospital Serdang, Malaysia. A total of 100 bone marrow trephine (BMT) images for each MPN type were captured from raw sample slides using a digital microscope from the Olympus model (BX41 Dual head microscope) as shown in Fig. 3. Different types of lens were used (x10, x20, and x40) to obtain different levels of magnifying details of the BMT images.

The number of slides provided by the pathologist and the total number of images captured for each class are listed in Table 2.

**Table 2:** Number of slides provided by the pathologist and the number of images captured.

No	Type of MPN	Number of slides	Number of images
1.	ET	4 slides	100 images
2.	PV	4 slides	100 images
3.	MF	3 slides	100 images

### 3.2 Data cleaning and data checking

All data collected was labelled accordingly, followed by a parallel discussion with the pathologist to verify the originality preservation of the bone marrow images and to confirm the region of interest in the captured images. Images that fulfilled the criteria were kept while those without the artefact and region of interest were removed from the dataset.

Bone marrow biopsy image gathered from patients diagnosed with three types of Ph-negative MPN was established as a basic requirement for the inclusion criteria; whereas, the exclusion criteria were applied for any evolved MPN. Additionally, the data collected was not attributed to time, period, or any geographic location.

## 4 Limitations

This imagery dataset has a limited sample size due to the rare nature of the MPN disease and the availability of a single source of data collection. Besides, the bone marrow morphology may not be presented for all relative frequencies of features in the dataset.

## Ethics statement

This data collection did not involve any clinical experiment or direct involvement of patients.

No informed consent was obtained specifically for the bone marrow biopsy procedure. Patients were verbally informed about the collection of samples during their clinic visits. The verbal consent was documented by the doctor in charge in the patients’ notes before the procedure.

Ethical approval for data collection was obtained from the Ministry of Health, Malaysia with reference number NMRR-18-4023-42507 (IIR).

## CRediT authorship contribution statement

**Umi Kalsom Mohamad Yusof:** Conceptualization, Methodology, Software, Validation, Formal analysis, Investigation, Writing – original draft, Visualization. **Syamsiah Mashohor:** Supervision, Project administration, Conceptualization, Methodology, Writing – review & editing. **Marsyita Hanafi:** Supervision, Project administration, Conceptualization, Methodology. **Sabariah Md Noor:** Supervision, Project administration, Conceptualization, Methodology, Validation. **Norsafina Zainal:** Validation.

## Declaration of Competing Interest

The authors declare that they have no known competing financial interests or personal relationships that could have appeared to influence the work reported in this paper.

## Data Availability

Histopathology Imagery Dataset of Ph-Negative Myeloproliferative Neoplasm.v2 (Original data) (Mendeley Data)
